# Balancing Act of the Intestinal Antimicrobial Proteins on Gut Microbiota and Health

**DOI:** 10.1007/s12275-024-00122-3

**Published:** 2024-04-17

**Authors:** Ye Eun Ra, Ye-Ji Bang

**Affiliations:** 1 Department of Biomedical Sciences, Seoul National University College of Medicine, Seoul, 03080 Republic of Korea; 2 Department of Microbiology and Immunology, Seoul National University College of Medicine, Seoul, 03080 Republic of Korea; 3 Institute of Infectious Diseases, Seoul National University College of Medicine, Seoul, 03080 Republic of Korea

**Keywords:** Gut microbiota, Antimicrobial proteins, Intestinal epithelial cells, Intestinal immunity, Intestinal health

## Abstract

The human gut houses a diverse and dynamic microbiome critical for digestion, metabolism, and immune development, exerting profound effects on human health. However, these microorganisms pose a potential threat by breaching the gut barrier, entering host tissues, and triggering infections, uncontrolled inflammation, and even sepsis. The intestinal epithelial cells form the primary defense, acting as a frontline barrier against microbial invasion. Antimicrobial proteins (AMPs), produced by these cells, serve as innate immune effectors that regulate the gut microbiome by directly killing or inhibiting microbes. Abnormal AMP production, whether insufficient or excessive, can disturb the microbiome equilibrium, contributing to various intestinal diseases. This review delves into the complex interactions between AMPs and the gut microbiota and sheds light on the role of AMPs in governing host-microbiota interactions. We discuss the function and mechanisms of action of AMPs, their regulation by the gut microbiota, microbial evasion strategies, and the consequences of AMP dysregulation in disease. Understanding these complex interactions between AMPs and the gut microbiota is crucial for developing strategies to enhance immune responses and combat infections within the gut microbiota. Ongoing research continues to uncover novel aspects of this intricate relationship, deepening our understanding of the factors shaping gut health. This knowledge has the potential to revolutionize therapeutic interventions, offering enhanced treatments for a wide range of gut-related diseases.

## Introduction

The human gastrointestinal (GI) tract houses trillions of microorganisms known as microbiota, which serve as a key regulator of numerous essential functions, such as digestion, metabolism, and immune development, and are thus crucial in maintaining human health (Basolo et al., 2020; Sender et al., 2016). However, when these microbes breach the gut barrier and enter host tissues, they can lead to infections and serious health issues, such as inflammation and sepsis (Rogers et al., 2023; Ruff et al., 2020). Intestinal epithelial cells are located at the key interface in between host and microbes, serving as both a physical and chemical barrier. They play a pivotal role in defending against microbial invasion and fostering a beneficial host-microbe relationship (Hooper et al., 2012). Antimicrobial proteins (AMPs) are key innate immune effectors that are produced by intestinal epithelial cells and regulate the gut microbiota by directly eliminating or inhibiting microbes (Bevins & Salzman, 2011; Vaishnava et al., 2008). Dysregulation of AMP production, whether it be insufficient or excessive, can disrupt the microbiota’s balance, contributing to various intestinal diseases (Mukherjee & Hooper, 2015). Therefore, understanding the regulation of AMP production and activities in the epithelium, along with its impact on the intricate interplay with the gut microbiota, is crucial for gaining insights into and addressing gut-related diseases effectively. This review highlights our current knowledge of the roles and importance of regulation and functions of AMPs on the host-microbe interactions.

## Gut Microbiota

The gut microbiota encompasses a diverse population of microorganisms, including bacteria, fungi, archaea, viruses, and protozoa, residing in the GI tract. This intricate ecosystem is crucial for various essential host functions. The gut microbiome enhances the host’s capacity to extract energy from ingested nutrients, and the metabolites and byproducts produced by microbiota such as short-chain fatty acids (SCFAs), secondary bile acids, and lipopolysaccharides (LPS), regulate appetite, nutrient uptake, gut motility and energy expenditure (Carmody & Bisanz, 2023; Hayes et al., 2018; Turnbaugh et al., 2009). Also, the gut microbiota regulates the development and homeostasis of both innate and adaptive immune systems. Dysregulation in the activities, abundance or anatomical localization of the microbiota may lead to loss of tolerance, resulting in development of inflammation and immune-mediated pathology (Renz & Skevaki, 2021; Ruff et al., 2020).

In recent decades, substantial research has unveiled intricate links between the gut microbiome and a diverse spectrum of diseases including cancer, diabetes, and neurological disorders (Holmes et al., 2020). Microbiota-synthesized metabolites influence the onset of disease symptoms. For example, the direct impact of plasma trimethylamine-N-oxide (TMAO), produced by the microbial fermentation of choline and carnitine, on cardiovascular disease is well known (Wang et al., 2015). It has also been reported that microbial tryptophan metabolites such as indole can impact the development and progression of diseases such as inflammatory bowel disease (IBD), tumors, obesity and metabolic syndrome, neurological diseases, infectious diseases, vascular inflammation and cardiovascular disease, and liver fibrosis (Su et al., 2022). Moreover, microbial dysregulation can lead to immune dysfunctions involving various gastrointestinal problems such as increased infection susceptibility, uncontrolled inflammation, and autoimmune development (Mousa et al., 2022). Therefore, it is crucial to maintain proper regulation of the gut microbiota for health, even though we have yet to fully grasp the complete spectrum of this regulation.

## Intestinal Epithelial Cells: The Primary Modulator of Host-microbe Crosstalk

Intestinal epithelial cells are pivotal in maintaining gut homeostasis through their roles in the establishment of a primary physical barrier, orchestration of immunity, modulation of the gut microbiota, and facilitation of immune communication. The intestinal epithelial cells delineate the boundary between the internal milieu of the body and the external environment of the intestinal lumen. This barrier serves as an indispensable line of defense by adeptly regulating selective permeability facilitating the absorption of vital nutrients, the expulsion of waste products, and the robust inhibition of potentially harmful pathogens and deleterious molecules (Horowitz et al., 2023). Also, epithelial cells sense nutrients and microbiota and critically orchestrate immune responses accordingly (Bang, 2023; Bang et al., 2021; Gattu et al., 2019; Haq et al., 2021; Hu et al., 2019).

**Fig. 1:**
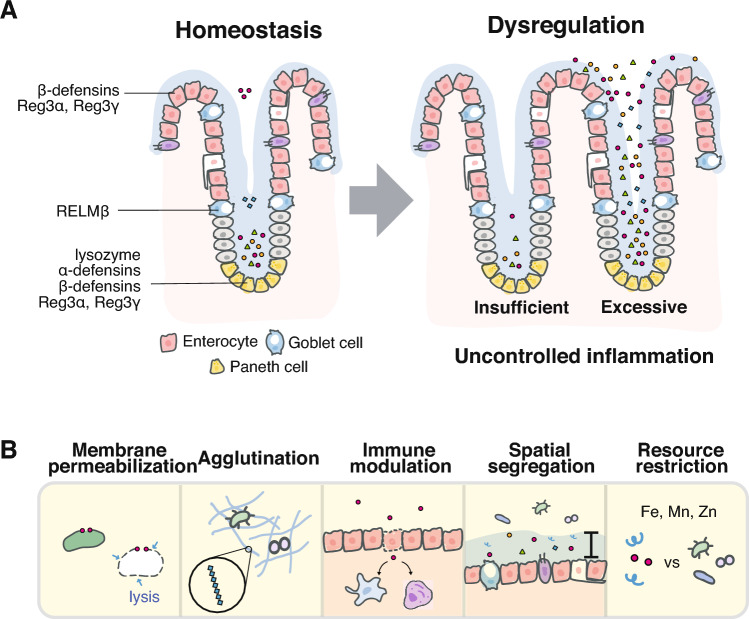
Antimicrobial proteins as key regulators of host-microbiota interactions. **A** Antimicrobial proteins (AMPs) are essential for gut homeostasis, the dysregulation of which, either insufficient or excessive, leads to uncontrolled inflammation. Examples of AMPs produced by distinct epithelial cells (enterocytes, goblet cells, Paneth cells) are indicated. **B** Mechanisms of AMPs in regulating microbiota. Many AMPs exhibit antimicrobial activity by disrupting bacterial membranes. Some, like HD6 and hBD1, agglutinate and trap bacteria. AMPs can directly modulate immune cell functions and spatially segregate microbiota from epithelium. AMPs can limit essential resources such as Fe, Mn, Zn, from bacteria, inhibiting bacterial growth

One important component of the host’s defense against external bacterial threats is the AMPs produced by the intestinal epithelial cells (Fig. 1). AMPs are the evolutionarily conserved innate immune effectors that exert remarkable control over the gut microbiome with the ability to directly kill microbes or impede their growth. AMPs exhibit significant structural and functional diversity as highlighted in Table 1 (Wang, 2014; Ragland and Criss 2017). Many small AMPs, which typically consist of fewer than 50 amino acids such as defensins and cathelicidins, possess a positive charge important for selectively interacting with anionic bacterial membranes rather than the zwitterionic membranes found in humans. Concurrently, the hydrophobic components of AMPs facilitate effective interactions with the hydrophobic interior of the bacteria cell membranes (Wang, 2014). AMPs control the penetration of commensal and pathogenic bacteria and are crucial for maintaining intestinal homeostasis at the host-microbial interface (Mukherjee & Hooper, 2015). In addition to their direct bacterial antimicrobial activity, AMPs also promote immune responses by attracting various immune cells, including neutrophils, eosinophils, mast cells, monocytes, and lymphocytes, to the site of infection (Davidson et al., 2004). Interestingly, microbial communities show distinct distributions in different regions of the gut. Similarly, AMPs also show specific spatial expression patterns within the gut (Castillo et al., 2019; Karlsson et al., 2008). Therefore, investigating how the biogeography of AMPs and gut bacteria fine-tune each other in the intestinal environment would be an interesting area of research.

**Table 1:** Characteristics and mechanisms of action of antimicrobial peptides and proteins

AMP	Structure	Representative genes	Source in GI tract	Mechanism	Target microbes	References
α-Defensin	Triple-stranded β-sheet, three disulfide bonds	HD5	Paneth cell	Membrane permeabilization by forming a dimer pore	G(−) G(+) Fungi Viruses	Ericksen et al. (2005); Porter et al. (1997); Selsted and Ouellette (2005)
		HD6	Paneth cell	Net-like structure formation to inhibit transmigration of microbiome	Capturing pathogens	
β-Defensin	Triple-stranded β-sheet, one α-helix four disulfide bonds	hBD1	Constitutive expression by IEC	Net-like structure formation to inhibit transmigration of microbiome	G(−) Viruses Fungi	Hoover et al. (2000); Nuding et al. (2009); Raschig et al. (2017); Selsted and Ouellette (2005)
		hBD2	Paneth cell	Membrane permeabilization based on electrostatic charge	G(−) Fungi Viruses	
		hBD3	Paneth cell	Perturbing the bacterial cell wall biosynthesis	G(−) G(+) Fungi Viruses	
Cathelicidins	Transition from a disordered structure to an alpha-helix structure	LL-37	Colon epithelial cell	Membrane permeabilization by forming toroidal pore	G(−) G(+) Fungi Viruses	Dürr et al. (2006); Iimura et al. (2005)
Lysozyme	Three-dimensional globular conformation	Lyz	Paneth cell	Enzymatic degradation of 1,4-β-glycosidic linkages of peptidoglycan Bacterial growth inhibition	G(−) G(+) Fungi Viruses	Ganz (2004); Peeters and Vantrappen (1975)
Lectin	Activation form with N-terminal prosegment removed	RegIIIα	Enterocyte Paneth cell	Membrane permeabilization by forming a hexamer pore	G(+)	Cash et al. (2006); Lehotzky et al. (2010); Mukherjee et al. (2014); Vaishnava et al. (2011)
		RegIIIγ	Enterocyte Paneth cell	Membrane permeabilization by forming a hexamer pore Spatial segregation	G(−) G(+)	
	Carbohydrate recognition domains	Galectin-1, 3, 9	Constitutive expression by IEC	Inflammasome activation	G(−) G(+) Fungi Viruses	Liu and Stowell (2023)
Resistin-like molecules (RELMs)	Cysteine-rich and cationic motifs	mRELMβ	Goblet cell	Membrane permeabilization by forming a pore Spatial segregation	G(−)	Propheter et al. (2017); Steppan et al. (2001)

These properties make AMPs the critical gatekeepers in the dynamic symbiotic relationship between the host and gut microbiota. Therefore, understanding the role of AMPs produced by epithelial cells is essential for developing strategies to enhance the immune response and effectively combat gut microbial infections.

## Antimicrobial Proteins—Roles and Mechanisms of Action

AMPs possess distinct properties that contribute uniquely to the dynamic defense mechanisms, playing multifaceted roles in maintaining microbial homeostasis (Table 1 and Fig. 1).

### a. Defensin

Defensins are small cationic peptides with wide-range antibacterial activity, characterized by a core β-sheet structure stabilized by intramolecular disulfide bonds. They bind to cell membranes, forming transmembrane pores, permeabilizing the membrane, and reducing bacterial viability. Detailed mechanistic models regarding defensin-mediated transmembrane pore formation are discussed elsewhere (Fu et al., 2023; Ganz, 2003).

The defensins produced by human and mouse epithelia are categorized into α-defensins and β-defensins (Selsted & Ouellette, 2005). Mouse α-defensins, often referred to as Cryptins, exhibit variants and subgroups based on the specific section of the small intestine they are associated with (Castillo et al., 2019). Human α-defensins, HD5 and HD6, secreted by Paneth cells, show different susceptibility to proteolytic digestion by matrix metalloproteinases. This differential degradation results in a variety of active antimicrobial fragments, contributing to an environment-dependent defense mechanism and microbial homeostasis (Ehmann et al., 2019). HD5 regulates the gut microbiota under the influence of salt concentration and pH, effectively targeting a variety of bacteria (Porter et al., 1997; Shimoda et al., 1995). HD6 shows less bactericidal activity at biological concentrations but is significant in agglutinating and isolating bacteria without directly killing them (Chu et al., 2012).

Human β-defensins (hBDs) are secreted by most leukocytes and other epithelial cells. hBD-1 is constitutively expressed, whereas hBD-2 expression is induced by IL-1α or bacterial infection, playing a crucial role in the innate immune response, offering protection against various pathogens, and serving as an inflammation marker (Cieślik et al., 2021; Niyonsaba et al., 2005; O’Neil et al., 2000). hBD-2 and hBD-3 showed strain-specific antibacterial activities rather than species-specific. Aerobic bacteria were more susceptible to hBD-2 and hBD-3 than anaerobic bacteria, suggesting that hBD-2 and hBD-3 have similar mechanisms but may utilize different pathways (Joly et al., 2004). The hBDs may also be involved in immune response by regulating the production of cytokines and chemokines, which will be discussed further in the section below.

### b. Cathelicidin

Cathelicidins, found across several mammalian species, are produced by various cells, including neutrophils and macrophages, in tissues such as the skin and respiratory and GI tracts (Chow et al., 2013). To exert antimicrobial properties, the peptides undergo proteolytic cleavage, transforming inactive precursors into active peptides that effectively combat microbial pathogens (Gudmundsson et al., 1996). The specific proteolytic enzyme involved can vary depending on the type of cathelicidin and the species in which it is found. In humans, for example, the only cathelicidin peptide, LL-37, is initially produced as an inactive precursor protein known as hCAP18, which is then cleaved by proteolytic enzymes, such as elastase in neutrophils and proteinase 3, to generate the active LL-37 peptide (Sørensen et al., 2001).

They work similarly to the positively charged defensins family. They can bind to bacterial membranes and disrupt them, induce the recruitment of immune cells, and even break down bacterial biofilms (Niyonsaba et al., 2002; Sochacki et al., 2011). These actions are effective against Gram-positive (G(+)) and Gram-negative (G(−)) bacteria, as well as fungal species. CRAMP, a cathelin-related antimicrobial peptide expressed in embryonic and adult mice, demonstrates potent antimicrobial effects against G(−) bacteria. Its expression in myeloid precursors and neutrophils suggests a non-oxidative mechanism for microbial killing in mice (Bals & Wilson, 2003; Gallo et al., 1997). While their exact location of activity can vary depending on the species and the specific cathelicidin, their overall function in the gut is centered around antimicrobial defense and maintaining intestinal homeostasis.

### c. Lysozyme

Lysozyme is stored in large intracellular granules within Paneth cells and is actively released in response to stimuli such as bacterial infection (Peeters & Vantrappen, 1975). The enzymatic activity of lysozyme exerts broad-spectrum antimicrobial activity because it targets structural components that are conserved across bacterial species. The enzyme is specialized in hydrolyzing the 1,4-β-glycosidic bond between N-acetylglucosamine and N-acetylmuramic acid in peptidoglycan. This activity makes lysozyme particularly effective against G(+) bacteria; unlike the protective outer membrane of G(−) bacteria, G(+) bacteria are more susceptible to the action of lysozyme due to their relatively easy access to the cell wall (Ganz, 2004).

Importantly, lysozyme can be secreted through a secretory autophagy and is constantly present in the intestinal lumen. This allows quick and efficient responses to bacterial invasion, overcoming destruction of the Golgi apparatus (Bel et al., 2017). The antimicrobial activity of lysozyme is significantly enhanced when it works in conjunction with other antimicrobial factors present, as shown by studies of the binding action of AMPs in human airway surface fluids (Singh et al., 2000), suggesting that lysozyme’s antimicrobial action may extend beyond a specific group of bacteria. Such a cooperative action would strengthen the gut’s defense mechanisms against a wide range of pathogenic microorganisms.

### d. Lectin

C-type lectins are a class of calcium-dependent carbohydrate-binding proteins involved in recognition and elimination of microorganisms. Antimicrobial C-type lectins have direct antimicrobial activity against G(+) and G(−) bacteria (Cash et al., 2006; Miki et al., 2012). RegIII lectins belong to a subgroup of the C-type lectin family and are expressed in the small intestine in response to microbial signaling. RegIIIα and RegIIIγ are produced by a variety of small intestinal epithelial cells, stored in enterocytes and Paneth cells, and then released into the lumen (Vaishnava et al., 2008). These proteins possess a strong affinity for peptidoglycan carbohydrates, a key component of bacterial cell walls, and form a membrane-permeabilizing oligomeric pore and kill bacteria (Cash et al., 2006; Lehotzky et al., 2010; Mukherjee et al., 2014). While its primary binding target is peptidoglycan, it can also engage with the carbohydrate components within LPS, enabling it to effectively target and eliminate G(−) bacteria (Miki et al., 2012; Stelter et al., 2011).

Galectins are lectins that specifically bind to glycans containing galactose residues found on glycoproteins and glycolipids. Galectins are known to be predominantly expressed on innate immune cells, but galectins-1, -3, and -9 are also highly expressed on intestinal epithelial cells (Stowell et al., 2010). In addition to regulating immune cells, galectin-9 has been shown to exhibit antimicrobial activity against microorganisms by binding directly to LPS on G(−) bacteria and acting as eat-me signal to control selective autophagy through regulation of the inflammasome (Wang et al., 2021). Galectins can directly interact with fungi, viruses, and parasites and induce cell death, which is described in detail in other reviews (Liu & Stowell, 2023).

### e. Resistin-Like Molecules (RELMs)

Resistin-like molecules (RELMs) are a family of proteins involved in a variety of biological processes, including inflammation, host defense, and metabolism. In rodents, this family consists of four proteins (RELM-α, -β, -γ, and resistin) (Pine et al., 2018). RELMα, RELMβ, and resistin have been reported to exhibit antibacterial activity by binding to negatively charged lipids and forming size-selective pores that permeate bacterial membranes, shaping the tissue microbiome (Harris et al., 2019; Propheter et al., 2017). Studies also suggest that RELM proteins promote immunity to various helminth and bacterial infections (Pine et al., 2018).

### f. Others—SPRR, sPLA2, Ang4, PYY

Antimicrobial activities of the small proline-rich proteins (SPRR) have recently been revealed, with their ability to directly bind to negatively charged lipids and to permeabilize the bacterial membranes (Hu et al., 2021). In the GI tract, SPRR2A is expressed in Paneth cells and goblet cells by type 2 cytokines, and defends against bacterial invasion during helminth infection (Hu et al., 2021). In the skin, SPRR proteins were induced by LPS and demonstrated the bactericidal activities conferring the protection against various skin pathogens such as methicillin-resistant *Staphylococcus aureus* and *Pseudomonas aeruginosa* (Zhang et al., 2022).

Mammalian secreted phospholipase A2 (sPLA2) possess the capability to selectively target and hydrolyze fatty acids and polar heads of phospholipid substrates, which is distinctive from other members of PLA2 superfamily (Pan et al., 2002). The enzyme is present in a variety of tissues and cells and performs unique functions in the body. In the GI tract, it is synthesized in Paneth cells and is thought to play a role in regulating the gut microbiome (Senegas-Balas et al., 1984). The subsequent review delves into a more comprehensive examination of the function of sPLA2-IIA (Murakami et al., 2023).

Angiogenin-4 (Ang4), is a protein expressed in Paneth cells that has been shown to have direct antimicrobial activity against a variety of microorganisms, including G(+) and G(−) bacteria, fungi, and viruses (Hooper et al., 2003). Ang4 belongs to the superfamily of RNases, enzymes that catalyze the degradation of RNA (Harder & Schröder, 2002), and it is still unclear whether RNase activity is involved in its bactericidal function.

Peptide YY (PYY), a full-length form specifically produced from Paneth cells, exhibits AMP activities against virulent *Candida albicans* hyphae but not the yeast form. This activity is attributed to its cationic property, enabling it to bind to the negatively charged membrane of hyphae (Pierre et al., 2023).

## Dynamic Interplay of AMP and Microbiota

### a. AMP’s Indirect Action on Microbiota

In addition to the direct microbial killing mechanisms described above, AMPs regulate microbial numbers in a variety of ways such as immune modulation, spatial isolation, and resource restrictions, which provides an effective defense strategy against microbial invasion (Fig. 1).

**Immune modulation:** AMP directly stimulates TLRs to modulate the activity of immune cells such as macrophages and neutrophils and release immune signaling molecules to prepare the defense system against microbes (Abreu, 2010). This fine-tuning allows for a delicate balance between protecting against pathogens and tolerating beneficial commensal bacteria. For example, murine BD-2 (Defb2) acts as an endogenous signaling ligand for the microbial pattern recognition receptor TLR4 and acts directly on immature dendritic cells to induce dendritic cell maturation (Biragyn et al., 2002). Monocytes also differentiate into dendritic cells in the presence of LL-37 and vitamin D, enhancing the host’s primary T cell immune response (Greiller & Martineau, 2015). Cathelicidins, found in leukocytes and epithelial tissues, not only play a role in killing bacteria but also engage in diverse biological processes such as inflammation, cell proliferation and migration, immune modulation, wound healing, angiogenesis, and the secretion of cytokines and histamine (Bals & Wilson, 2003; Gallo et al., 1997).

**Spatial segregation:** AMPs regulate spatial segregation between the microbiota and the intestinal epithelium to promote host-bacteria mutualism. In the small intestine, where nutrient absorption occurs primarily, mucus is loose and permeable, yet its spatial segregation role is maintained by AMPs. Typically, RegIIIγ acts to physically separate the microbiome from the SI epithelium by a ~50 µm zone (Vaishnava et al., 2008). MyD88-dependent TLR activation induces RegIIIγ expression to control the number of mucosal bacteria and maintain host-microbe spatial segregation (Vaishnava et al., 2011). Although it cannot regulate the total number of bacteria, it does regulate the number of mucosal bacteria, physically preventing microbial invasion. Similarly, RELMβ also promotes spatial segregation of the microbial community and colonic epithelium. In RELMβ-deficient mice, proteobacteria reside in the mucus layer and invade mucosal tissue (Propheter et al., 2017). It is notable that the distortion of the mucosal immune response along the length of the gut is associated with a highly heterogeneous distribution pattern of the mucosal microbiota, suggesting that the mucosa regulates the mutual co-operation and functional stability of the intestinal ecosystem (Zhang et al., 2014). Additionally, differences in the size and physical properties of AMPs create variations in permeability into the mucus layer (Mergaert, 2018), suggesting another role for the mucosa in regulating the activity of antimicrobial peptides.

**Resource restriction:** There are several proteins that exert indirect antimicrobial effects through limiting essential resources from the microbes. Since iron is vital for the growth of nearly all bacteria, hosts employ strategies to restrict its availability. For instance, lactoferrin, an iron-binding protein, inhibits the growth of G(−) bacteria by sequestering iron, thereby depriving them of this essential element (Farnaud & Evans, 2003). Another protein, calprotectin, is integral to resource competition as it impedes the superoxide defense mechanism of manganese-dependent bacteria. By binding with manganese or zinc at their metal-binding sites, calprotectin increases intracellular levels of superoxide, thereby augmenting antimicrobial activity (Kehl-Fie et al., 2011). Lipocalin, on the other hand, restricts bacterial growth by inhibiting the enterochelin pathway, which is a route for bacteria to acquire iron (Flo et al., 2004). Through this resource limitation, the interaction between bacteria and hosts is regulated, providing another perspective on how the host’s immune system modulates its response to infections.

### b. Bacterial Strategies to Evade AMPs’ Action

Research has demonstrated various strategies by which bacteria exhibit resistance to AMPs. For example, some bacteria produce proteases and trapping proteins; others alter their cell surface charge, modify membrane fluidity, and activate efflux pumps. Additionally, bacteria utilize biofilms and exopolymers for protection and develop sensing systems through the selective gene expression.

**Removal of AMPs:** Proteases, particularly trypsin and chymotrypsin, are known to be an insurmountable obstacle for AMPs (Vlieghe et al., 2010), and certain bacteria produce proteases to develop resistance to AMPs. Bacteria and viruses, including *Pseudomonas aeruginosa* and *Haemophilus influenzae*, common causes of infections, are known to resist many AMPs by producing proteases that degrade and inactivate the structure of AMPs (Mason et al., 2005; Schmidtchen et al., 2002). To overcome this, recent developments include AMP antibiotics designed with reduced susceptibility to proteases.

Certain bacteria enhance the expression of efflux pumps. These pumps actively expel AMPs from the microbial cell, thereby reducing its intracellular concentration. This pump-mediated efflux acts as a defense mechanism to expel AMP before it can exert its antimicrobial effects. Examples of ABC transporters, capable of transporting a wide variety of substrates across biological membrane, involved in AMP efflux include the CPR (cationic antimicrobial peptide resistance) system of *Clostridioides difficile* and the AnrAB transporter of *Listeria monocytogenes* (Collins et al., 2010; Suárez et al., 2013; Clemens et al., 2018).

**Structural changes of cell membrane:** To evade the antimicrobial activity of AMPs, microorganisms alter their basic structure. Some microorganisms reduce the negatively charged components of the cell membrane, decreasing the probability of cationic AMPs binding to it. Others modify the cell wall peptidoglycan to block the enzymatic activity of lysozymes and specifically interfere with IL-1β secretion (Shimada et al., 2010).

One interesting strategy involves incorporating carotenoids into the bacterial membrane. Carotenoids, pigmented compounds found in various organisms including bacteria, are well-known for their antioxidant properties (Harris et al., 2019). Recent studies have shown that certain bacteria use carotenoids to mitigate the harmful effects of AMPs on their cell membranes. Carotenoids can absorb and scavenge reactive oxygen species produced during the interaction between AMPs and bacterial cell membranes. This mitigation of oxidative stress helps the bacteria maintain the integrity of their membranes and reduce the extent of damage caused by AMPs. By employing this strategy, bacteria can become more resilient to AMP-mediated death, enabling them to persist within the host’s environment.

**Physical protection from AMPs:** Some bacteria produce trapping exopolymers and proteins to prevent AMPs from reaching their target. Several bacterial anionic capsule polysaccharides bind to AMPs and block their activities against microbes (Campos et al., 2004; Llobet et al., 2008). Interestingly, bacteria enhance the release of CPS, which in turn protects them from AMPs (Llobet et al., 2008). Similarly, the anionic nature of bacterial biofilm components, such as extracellular polysaccharides, enables the trapping of positive AMPs, preventing their action (Yasir et al., 2018). On the other hand, *Staphylococcus aureus* has been shown to produce exoprotein called staphylokinase that binds to and neutralizes α-defensins (Jin et al., 2004). These extracellular materials can act as a physical barrier and prevent AMPs from reaching their target.

## Regulation of AMP Production in Disease and Health

### a. Microbiota and AMP Production

Endogenous AMPs exhibit diverse expression patterns. Some, like members of the lysozyme, sPLA2, and β-defensin protein families, are constitutively active regardless of microbial presence, while others respond to bacterial stimuli. Cryptdin-related sequences (CRS) peptide family components as well as human β-defensins, including hBD2, demonstrate adaptability to microbial cues. In germ-free mice, minimal expression of Ang4 and RegIIIγ increases upon microbial colonization, indicating responsiveness to microbes. While further research is needed to unravel this regulatory network, the following section discusses some of the uncovered mechanisms.

**Toll-like receptor activation:** Paneth cells possess the ability to independently detect intestinal bacteria through MyD88-dependent TLR activation (Vaishnava et al., 2011). This detection leads to the upregulation of several antimicrobial factors, including RegIIIγ (Cash et al., 2006; Vaishnava et al., 2011). At the same time, bacterial substances such as LPS or flagella is transmitted through TLRs on dendritic cells, which in turn, promotes the production of the pro-inflammatory cytokine IL-23. Subsequently, IL-23 prompts innate lymphoid cells to express IL-22, with IL-22 playing an important role in driving the transcriptional upregulation of RegIIIγ (Sanos et al., 2009; Valeri & Raffatellu, 2016). Also, bacterial lipoproteins are shown to induce hBD-2 from human lung epithelial cells through TLR2 (Birchler et al., 2001).

**Short chain fatty acids:** Studies suggest that gut microbiota-derived SCFAs induce AMP production. Butyrate induces the production of RegIIIγ and β-defensins in GPR43-dependent manner (Zhao et al., 2018), and LL-37 homologue CAP-18 (Raqib et al., 2006), while propionate also induces the production of RegIIIs (Bajic et al., 2020).

**Cytokines:** In the context of AMP regulation of bacteria through immunity, microorganisms can influence the expression of AMPs by affecting cytokine productions (Diamond et al., 2009). Particularly, it is reported that RELMs are expressed by M2 macrophages activated during parasitic infections and allergies. According to this research, RELMβ expression is also induced by type 2 cytokines such as IL-4 and IL-13 (Keegan et al., 2021; Oeser et al., 2015). Additionally, IL-13 stimulates the transcriptional upregulation of SPRR2A expressed in lung epithelial cells (Zimmermann et al., 2005).

The discovery that interferon-gamma (IFN-γ) induces the expression of cathelicidin in macrophages, enhancing the antimicrobial peptide cathelicidin’s activity against *Mycobacterium tuberculosis*. Likewise, IL-1β induces the expression of hBD-2 in keratinocytes, augmenting the activity of the antimicrobial peptide hBD-2 against *Staphylococcus aureus* (Cieślik et al., 2021; Niyonsaba et al., 2005).

### b. AMP Dysregulation and Disease

Interplay between AMPs and the gut microbiota is essential for maintaining gut health, and dysregulation of this intricate balance can have significant implications for disease development. Dysregulation in AMP expression and function have been associated with a range of gut-related disorders, such as inflammatory bowel disease (IBD) (Table 2). IBD encompasses chronic inflammatory conditions such as Crohn’s disease and ulcerative colitis, characterized by excessive immune responses within GI tract. Studies have demonstrated alterations in the expression of various AMPs in individuals with IBD. AMP dysregulation in the context of IBD is a consequence of inflammation, potentially contributing to its chronicity (Arijs et al., 2009). It appears that dysregulation in the expression and processing of AMPs represents a pivotal mechanism in IBD (Aldhous et al., 2009). Additionally, IBD patients exhibit an abnormal presence of lysozyme-secreting Paneth cells in the distal colon, characterized by elevated lysozyme levels (Dronfield & Langman, 1975; van der Sluys Veer et al., 1998). This is further evidenced by significantly higher concentrations of lysozyme observed in the stool and serum of IBD patients. Nevertheless, the precise role of lysozyme dysregulation in IBD’s pathogenesis remains incompletely elucidated. Specifically, a low copy number of the HBD2 gene, coupled with diminished mRNA expression, is linked to susceptibility to colonic Crohn’s disease (Aldhous et al., 2009). Changes in cathelicidin expression due to vitamin D deficiency has also been suggested to impair innate immunity in patients with ulcerative colitis (Gubatan et al., 2020).

**Table 2:** Antimicrobial peptides and proteins in IBD

Type of IBD	Dysregulation of AMPs		Correlation with disease	References
UC	HD5, HD6	Excessive	Association	Noble et al. (2008)
	hBD2	Excessive	Contribution	Aldhous et al. (2009); Langhorst et al. (2009)
CD	Lysozyme	Excessive	Association, Contribution	Noble et al. (2008); VanDussen et al. (2014); Yu et al. (2020)
	HD5, HD6	Insufficient	Association	Wehkamp et al. (2005, 2007)
	hBD1-3	Insufficient	Association	Fellermann et al. (2006)
UC/CD	Lysozyme	Excessive	Contribution	van der Sluys Veer et al. (1998)
	Cathelicidin	Excessive	Association, Contribution	Gubatan et al. (2020); Tran et al. (2017)
	Galectin-1	Excessive	Association	Frol’ová et al. (2009)
	Galectin-3	Excessive	Association, Contribution	
	RegIIIγ	Excessive	Association	Ogawa et al. (2003); Jang et al. (2023)

## Concluding Remarks

The intricate interplay between the gut microbiota and antimicrobial proteins constitutes a dynamic dialogue that shapes the delicate equilibrium within the gut ecosystem. Through a multifaceted array of interactions, AMPs contribute to defending against invading pathogens, maintaining microbial diversity, and modulating immune responses. This review has provided a comprehensive exploration of the mechanisms governing the interactions between AMPs and the gut microbiota, providing insights into their roles in both health and disease.

Understanding the complex regulatory networks governing AMP expression, microbial resistance mechanisms, and the implication of dysregulation offers valuable insights into the intricate balance required for gut homeostasis. Furthermore, the challenges posed by microbial resistance to AMP-induced killing highlight the need for innovative strategies to enhance the efficacy of antimicrobial defense mechanisms. Leveraging this knowledge holds promise for developing novel therapeutic approaches targeting the gut ecosystem to promote health and prevent disease.

As we navigate the frontiers of gut-microbiota interactions, ongoing research will undoubtedly uncover new facets of this complex relationship. Unraveling the complex communications between the host and gut microbiota paves the way for a deeper understanding of the factors governing gut health. Ultimately, this knowledge has the potential to revolutionize our approach to therapeutic interventions, leading to improved treatments for a diverse range of gut-related disorders.
